# Type III Secretion Protein, PcrV, Impairs *Pseudomonas aeruginosa* Biofilm Formation by Increasing M1 Macrophage-Mediated Anti-bacterial Activities

**DOI:** 10.3389/fmicb.2020.01971

**Published:** 2020-08-13

**Authors:** Hua Yu, Junzhi Xiong, Jing Qiu, Xiaomei He, Halei Sheng, Qian Dai, Defeng Li, Rong Xin, Lu Jiang, Qiaoqiao Li, Qian Chen, Jin Peng, Maolin Wang, Xiancai Rao, Kebin Zhang

**Affiliations:** ^1^Central Laboratory, Xinqiao Hospital, Army Medical University, Chongqing, China; ^2^Department of Microbiology, College of Basic Medical Sciences, Army Medical University, Chongqing, China

**Keywords:** *Pseudomonas aeruginosa*, biofilm, PcrV, immune evasion, macrophage polarization, biofilm eradication

## Abstract

*Pseudomonas aeruginosa* biofilms employ a variety of strategies to hijack the host immune defense system to achieve chronic infection. However, the bacterial components that are involved in this process are not yet fully understood. PcrV, a needle tip protein of the *P. aeruginosa* type III secretion system (T3SS), was downregulated during *P. aeruginosa* biofilm infection. The impaired expression of the *P. aeruginosa pcrV* gene is associated with attenuated immune activation and an increased percentage of M2 macrophages following *P. aeruginosa* biofilm infection. Treatment with exogenous PcrV produced from *Escherichia coli* elevated tissue inflammation and the percentage of M1 macrophages, resulting in reduction in the biofilm burden. Further analyses demonstrated that the potential of PcrV to induce classically activated M1 macrophages as evidenced by the increased production of proinflammatory cytokines and anti-bacterial mediators, including inducible nitric oxide synthase (iNOS) and reactive oxygen species (ROS), as well as increased phagocytosis of bacteria. Mechanistically, PcrV-mediated promotion of macrophage M1 polarization and phagocytosis occurs through the activation of mitogen-activated protein kinases (MAPKs) and NF-κB signaling pathways. Collectively, these findings reveal a potential role of PcrV in skewing host immune defense to promote *P. aeruginosa* biofilm infection and provide new insights into the therapeutic strategies for *P. aeruginosa* biofilm infection.

## Introduction

*Pseudomonas aeruginosa*-mediated chronic infections are commonly associated with biofilm formation on host tissues and indwelling medical devices. Bacterial biofilm communities are encased in a self-produced extracellular matrix which precludes antibiotic penetration, which impedes eradication of biofilm bacteria by conventional antibiotic treatment. To combat biofilm infections, various strategies have been investigated, including utilization of antimicrobial peptides/lipids (Verderosa et al., 2019), quorum sensing inhibitors (Chang et al., 2019), and bacteriophages (Geredew Kifelew et al., 2019). In addition to the methods that directly target bacterial components, promising new strategies to control biofilm infection rely on improving host anti-bacterial immune responses, such as the induction of proinflammatory cytokines and antibacterial mediators, for instance, nitric oxide (NO) and reactive oxygen species (ROS), and the enhancement of phagocytosis activity (Campoccia and Mirzaei, 2019).

Macrophages act as the first line of defense against bacterial infection by secreting proinflammatory cytokines and bactericides, as well as by increasing phagocytotic activity against bacteria (Keewan and Naser, 2020). However, differences in the polarization of macrophage phenotypes determine the diverse efficacy in the eradication of bacteria. The classically activated M1 macrophages release large amounts of proinflammatory cytokines, such as tumor necrosis factor (TNFα), interleukin 6 (IL6), and IL12, and antibacterial mediators, such as NO, ROS, and reactive nitrogen species (RNS). As such, M1 macrophages mediate efficient phagocytosis and are actively involved in microbicidal action (Qian and Pollard, 2010; Shapouri-Moghaddam et al., 2018). Conversely, the alternatively activated M2 macrophages are characterized by enhanced production of anti-inflammatory cytokines, IL4 and IL10 and arginase. As such, M2 macrophages are involved in attenuating microbicidal activity (Panagi et al., 2019). Although M1 macrophages play critical roles in eliminating planktonic bacteria, studies have demonstrated that macrophages display an M2 phenotype following activation by *Staphylococcus aureus* biofilms (Thurlow et al., 2011), suggesting that biofilms play a role in inducing an anti-inflammatory activation of macrophages that benefits biofilm persistence. Conversely, treatment of biofilms with M1-activated macrophages indicates the potential importance for controlling biofilm infections. However, the issues regarding the bacterial elements that affect biofilm persistence following *P. aeruginosa* biofilm infection, as well as the efficacy of M1 macrophages against *P. aeruginosa* biofilm infections, have not yet been reported.

PcrV, which is a critical needle tip protein of the type III secretion system (T3SS) of *P. aeruginosa*, is an indispensable factor that allows the translocator protein PopB/D to form pores on the host cell membrane through which effector proteins translocate into host cells. In addition to this biological function, PcrV has been reported to possess a possible proinflammatory function (Wangdi et al., 2010). As a V-antigen, a PcrV-originated vaccine elicited a multifactorial immune response and conferred broad protection in an acute *P. aeruginosa* pneumonia model (Hamaoka et al., 2017; Wan et al., 2019), indicating the potential of PcrV for the treatment of bacterial biofilm infection through activation of the host immune response. Nevertheless, the role of PcrV in modulating macrophage polarization and improving the efficacy of *P. aeruginosa* biofilm eradication remains to be elucidated.

In this study, we demonstrated that *pcrV* gene expression is reduced during *P. aeruginosa* biofilm infection. Injection of PcrV into the milieu surrounding the biofilm-infected tissues induced a proinflammatory response with increased infiltration of M1-polarized macrophages, decreasing the biofilm burden. Further analyses demonstrated that PcrV promotes biofilm eradication, macrophage M1 polarization, and phagocytosis *via* the mitogen-activated protein kinases (MAPKs) and NF-κB signaling pathways. Taken together, these results suggest that decreased expression of PcrV during *P. aeruginosa* biofilm infection promotes biofilm persistence and provide novel clues into the therapeutic strategies against *P. aeruginosa* biofilm infection.

## Materials and Methods

### Mice and Ethics Statement

Male mice on a C57BL/6 background were purchased from biocytogen CO., Ltd. (Beijing, China). Animal experiments were conducted according to the experimental animal guidelines of the Army Medical University of China.

### Expression and Purification of PcrV Protein

*Pseudonomas aeruginosa* PcrV gene was cloned into pQE31 (Qiagen, Germany), which introduces an N-terminal fusion of the protein to a His_6_ tag. *Escherichia coli* JM109 strain carrying expression plasmids was propagated in LB medium containing 100 μg/ml ampicillin until the OD_600_ nm reached 0.5 and was induced using 0.5 mM isopropyl-β-D-thiogalactopyranoside (IPTG) at 37°C for 4 h. Cells were harvested and resuspended in phosphate buffered saline (PBS) and lysed by sonication. The fusion protein was purified by affinity chromatography using His-Trap HP (GE healthcare, Germany). Endotoxin was removed from PcrV by using Detoxi-Gel endotoxin removing gel (Thermo fisher, USA) following the manufacturer’s instructions.

### Isolation and Induction of BMDMs

Tibias and femurs from the euthanized C57BL/6 mice were excised, and marrow cells were washed out with a 25-gauge needle attached to a 5 ml-syringe. Cells were cultured in 10% FBS/dulbecco’s modified eagle medium (DMEM), penicillin, streptomycin, and 50 ng/ml recombinant murine macrophage colony stimulating factor (M-CSF) at 37°C and 5% CO_2_ for 3 days. At day 4, the medium was replaced, and cells were cultured at same condition for an additional 3 days.

### Mouse Model of *P. aeruginosa* Catheter-Associated Biofilm Infection

A mouse model of catheter-associated biofilm infection was established as described previously with some modifications (Thurlow et al., 2011; Hanke et al., 2013). Briefly, a sterile 1 cm intravenous catheter was implanted subcutaneously into the flank of mice under pentobarbital sodium anesthesia. A suspension (20 μl) of log-phase PAO1 (1 × 10^5^ colony forming unit, CFU) was injected through the skin into the catheter lumen. Biofilm formation was monitored throughout the course of infection, and mice were sacrificed on days 2, 5, and 8 post-infection. For scanning electron microscopy (SEM) analysis, biofilms on catheters were fixed and dehydrated according to a standard SEM protocol and were observed under a Crossbeam 340 SEM (Carl Zeiss, Germany). Tissues surrounding infected catheters were homogenized and weighed after freezing in liquid nitrogen. The bacterial burdens of catheters and surrounding tissues were enumerated using *P. aeruginosa* isolation agar (PIA) plates.

### Macrophage Administration Into Biofilm Infections *in vivo*

To determine the efficacy of biofilm clearance by differentially polarized macrophages, 10^6^ non-activated or PcrV-activated bone marrow-derived macrophages (BMDMs) were subcutaneously injected at the sites surrounding infected catheters on days 5, 6, and 7 post-infection. The infected catheters and surrounding tissues were harvested on day 8 for subsequent analysis.

### Generation of *P. aeruginosa* Static Biofilms *in vitro*

Static biofilms were generated as previously described (Thurlow et al., 2011) with minor modifications. Briefly, sterile 24-well cell culture plates were treated with 20% human plasma in sterile carbonate-bicarbonate buffer (pH 9.6) overnight at 4°C to facilitate bacterial attachment. The PAO1 strain was cultured overnight at 37°C with shaking in DMEM supplemented with 10% FBS. The bacterial culture was adjusted to an initial OD_600_ of 0.05 and then incubated in the plasma pre-coated plate at 37°C under static aerobic condition for 3 days. Medium was carefully replenished every 24 h to prevent disruption of the biofilm structure.

### Phagocytosis Assay

The phagocytic efficacy of macrophages against planktonic bacteria or biofilms was evaluated according to a previously described method with modifications (Thurlow et al., 2011). Briefly, planktonic bacteria or biofilms were co-cultured for 30 min with RAW264.7 cells at a ratio of 1:10 at 37°C under 5% CO_2_. Cells were then treated with gentamicin (final concentration 400 μg/ml) for 2 h at 37°C under 5% CO_2_ to remove extracellular bacteria. For immunofluorescence staining, the cytoskeleton and bacteria were visualized by phalloidin (red) and anti-PAO1 antibody (green), respectively. For evaluation of the intracellular bacteria, cells were lyzed with 0.5% TritonX-100/PBS and enumerated on PIA plates.

### Western Blot

Cell pellets were lysed using RIPA buffer (Beyotime, China) supplemented with protease inhibitor cocktail (Roche, USA). Equal amounts of proteins were separated on 10% SDS-PAGE and then transferred electrophoretically to PVDF membranes (Millipore, USA). The membrane was blocked using 5% skim milk in TBST at real time (RT) for 1 h. Then, the membrane was incubated with the appropriate first antibody at 4°C overnight and horseradish peroxidase-conjugated secondary antibody at RT for 1 h.

### Real-Time Quantitative PCR

Total RNA extraction and reverse transcription to cDNA were performed according to the manufacturer’s instructions. Quantitative PCR was performed using an ABI 7500 RT PCR system (Applied Biosystems, Germany). For macrophages, the relative gene expression levels of *cd11c*, *inos*, *ptgs2*, *cd206*, and *pparγ* were normalized to GAPDH. For PAO1 strain, the relative gene expression level of *ndk* was normalized to *gyrB*, *rpoD*, or *rplU* gene. The primers used in this study were provided in Supplementary Table S1.

### Enzyme-Linked Immunosorbent Assay

Supernatants of stimulated macrophages or biofilm infected tissues were assessed for their levels of TNFα, IL 12 p40/p70, and IL6 by using the sandwich enzyme-linked immunosorbent assay (ELISA) kits (BD biosciences, USA) according to the manufacturer’s instructions.

### Measurement of Intracellular ROS

Macrophages were harvested and incubated with 2'7'-dichlorodihydrofluorescein diacetate (H2DCFDA; Santa Cruz, USA) dye in DMEM medium at a final concentration of 5 μM for 30 min at RT. Intracellular ROS were measured by flow cytometry.

### Statistical Analysis

Data were expressed as means ± standard errors of the means (SEM). The statistical analysis was carried out with GraphPad (GraphPad Software Inc., San Diego, CA). Data were analyzed by unpaired *t* test when comparing two groups and one-way analysis of variance (ANOVA) with Tukey’s multiple-comparison test for multiple groups. A value of *p* < 0.05 was considered statistically significant.

## Results

### *P. aeruginosa* Biofilm Persistence Is Associated With Attenuated Activation of Host Proinflammtory Responses and Decreased Bacterial Eradication Ability

**Figure 1:**
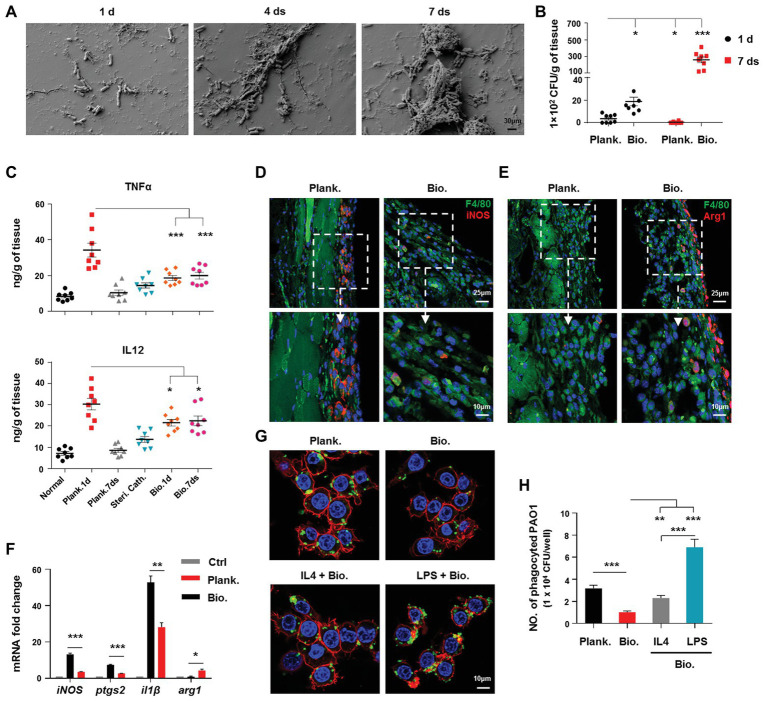
*Pseudomonas aeruginosa* biofilm persistence is associated with attenuated activation of host proinflammtory responses and decreased bacterial eradication ability, whereas increased programming of macrophages toward M2 phenotype. **(A)** *P. aeruginosa* biofilm formation on catheters was observed by scanning electron microscopy (SEM; 5000X magnification). Bacterial loads **(B)**, production of tumor necrosis factor (TNFα) and IL12 p40/70 **(C)**, and F4/80^+^/iNOS^+^ and F4/80^+^/Arg1^+^ macrophages (**D**,**E**) in planktonic *P. aeruginosa* (1 day)‐ or catheter biofilm (7 days)-infected tissues were determined by colony forming unit (CFU) enumeration, enzyme-linked immunosorbent assay (ELISA), and immunofluorescence staining, respectively. For immunofluorescence staining, M1 macrophages were counterstained with fluorescein isothiocyanate (FITC)-conjugated anti-F4/80 and AF647-conjugated anti-inducible nitric oxide synthase (iNOS) antibodies. M2 macrophages were counterstained with FITC-conjugated anti-F4/80 and AF647-conjugated anti-Arg1 antibodies; cellular nuclei were stained with 4′,6-diamidino-2-phenylindole (DAPI). The static *P. aeruginosa* biofilms were co-cultured with Raw264.7 at 37°C, with 5% CO_2_ for 3 h (MOI = 10), and relative gene expression levels were analyzed by real-time quantitative PCR (RT-qPCR) using mouse GAPDH as a reference gene **(F)**. To generate M1 and M2-polarized macrophages, Raw264.7 cells were pretreated with 100 ng/ml lipopolysaccharide (LPS) + 50 ng/ml IFNγ (named as LPS group) or 20 ng/ml IL4 for 6 h, respectively. The cells were then co-cultured with static *P. aeruginosa* biofilms at a ratio of 1:10 at 37°C, 5% CO_2_ for 30 min. Phagocytosis was detected by immunofluorescence staining **(G)** and CFU enumeration **(H)**. Cytoskeleton was labeled with phalloidin (red); PAO1 was visualized by FITC anti-PAO1 antibody (green); cellular nuclei were stained with DAPI (blue). Error bars indicate the means ± standard errors of the means (SEM). An unpaired Student’s *t* test was used for statistical analysis (**B**,**C**,**F**,**H**). ^*^p < 0.05; ^**^p < 0.01; ^***^p < 0.001. Planktonic (Plank.), biofilm (Bio.), sterile catheter (steri cath).

To evaluate the condition of the host immunity and bacterial killing efficacy in response to a *P. aeruginosa* biofilm, we used a mouse model of catheter-associated biofilms to mimic bacterial biofilm formation on medical devices in humans (Hanke et al., 2013). Bacterial biofilm formation involves a transformation from an immature to a mature structure involving bacterial surface attachment and bacterial colony formation in which bacteria are surrounded by matrix materials. In this study, we primarily observed the different stages of *P. aeruginosa* biofilm formation on catheters by SEM. Examination of the catheter lumen by SEM revealed that an immature biofilm was formed with few extracellular matrix-encompassed bacteria within 4 days of the initial infection (Figure 1A). With the extension of time post-infection (7 days), a mature biofilm was formed with a contiguous bacterial layer in which extracellular matrix and interior holes were found (Figure 1A), suggesting the successful establishment of a mature *P. aeruginosa* biofilm.

To compare the host immune responses and bacterial eliminating efficacy between *P. aeruginosa* biofilms and planktonic bacteria-associated infections, we analyzed the production of inflammation-related cytokines, arginase, and inducible nitric oxide synthase (iNOS), as well as the bacterial burdens in the tissues surrounding the site of the infection. At 1 day post-infection, bacterial loads in planktonic bacteria-infected tissues were lower than biofilm bacteria-infected group (Figure 1B; *p* < 0.05). At 7 days post-infection, the planktonic bacteria were almost eradicated, while the bacterial loads in the biofilm remained high (Figure 1B). In contrast to the decreased bacterial loads, the levels of the proinflammatory cytokines, TNFα (Figure 1C; *p* < 0.001) and IL12 p40/70 (Figure 1C; *p* < 0.05), and the percentages of iNOS^+^ cells were higher in planktonic bacteria-infected tissues (1 day post-infection) than those in biofilm-infected tissues (Supplementary Figure S1). Arg1, which is involved in activating anti-inflammatory responses, was also higher in biofilm-infected tissues than in planktonic bacteria-infected tissues (Supplementary Figure S1), suggesting a lower ability of *P. aeruginosa* biofilms to activate proinflammatory responses to infection.

Phagocytosis by phagocytes, such as macrophages, accelerates the clearance of bacteria following infection; therefore, we compared the phagocytotic ability of macrophages against *P. aeruginosa* in the planktonic and biofilm forms *in vitro*. The results showed that the ability of macrophages to phagocytose biofilm bacteria was inferior to that observed for planktonic bacteria (Figures 1G,H; *p* < 0.001). Collectively, these results indicated that *P. aeruginosa* biofilms enhance bacterial chronic infection by circumventing the host immune response and anti-bacterial activities.

### *P. aeruginosa* Biofilm-Mediated Programming of Macrophages Toward an M2 Phenotype Attenuates Immune Activation, and Promotes Biofilm Persistence

Based on the above results, we further investigated the association of the impaired activation of host immune responses to *P. aeruginosa* biofilm infection with alternatively activated M2 macrophages. Result revealed that a higher percentage of F4/80^+^/Arg1^+^ macrophages and a lower percentage of F4/80^+^/iNOS^+^ macrophages in biofilm-infected tissues (7 days post-infection) than those in planktonic bacteria-infected tissues (1 day post-infection; Figures 1D,E), reflecting a less M1-like phenotype of macrophages under a biofilm-associated infection. To further verify this phenomenon, we analyzed the gene expression profiles of Raw264.7 cells infected with *P. aeruginosa* planktonic bacteria or biofilms *in vitro*. In accordance with our *in vivo* findings, macrophages associated with the biofilm infection exhibited decreased expression of proinflammation-related genes, including *inos*, *il1β*, and *ptgs2*, compared with those associated with planktonic bacterial infection (Figure 1F), whereas the expression of *arg1* was increased (Figure 1F; *p* < 0.05).

The decreased phagocytic ability of M2 macrophages skewed by bacterial biofilms is also responsible for the delayed bacterial clearance and biofilm persistence. To this end, we analyzed the phagocytic ability of differentially polarized macrophages against *P. aeruginosa* biofilms. Although, lipopolysaccharide (LPS)‐ or IL4-primed M1 or M2 macrophages both displayed higher phagocytic ability than non-activated macrophages (Figures 1G,H), the M1 macrophages exhibited a superior ability for phagocytosis of biofilm bacteria than M2 macrophages (Figures 1G,H; *p* < 0.001). Collectively, these results suggested that *P. aeruginosa* biofilm-mediated skewing of macrophages toward the M2 phenotype enhances biofilm persistence.

### PcrV Production Is Attenuated in Chronic *P. aeruginosa* Biofilm Infection, and the Addition of Exogenous PcrV Accelerates Macrophage-Mediated *P. aeruginosa* Clearance in Biofilms

**Figure 2:**
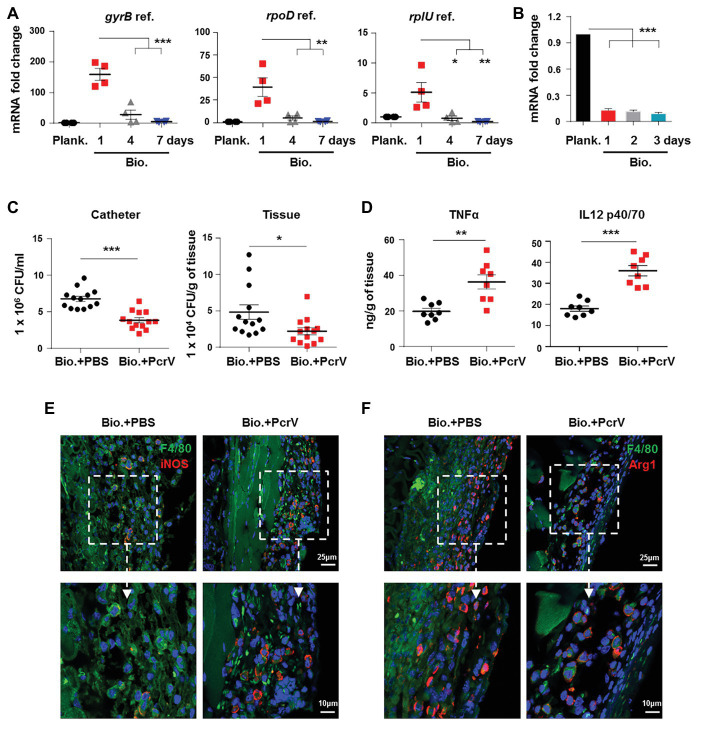
Decreased expression of *P. aeruginosa* PcrV gene following biofilm infection promotes biofilm persistence. **(A)** *P. aeruginosa*-infected catheters were subcutaneously implanted into C57BL/6 mice, and catheters were harvested after infection for 1, 4, and 7 days. The *pcrV* gene expression in biofilm catheter or planktonic bacteria harvested before infection was analyzed by RT-qPCR. *P. aeruginosa gyrB*, *rpoD*, and *rplU* genes were used as reference genes (abbreviated as ref.). **(B)** The *pcrV* gene expression in *P. aeruginosa* static biofilms or planktonic bacteria was analyzed by RT-qPCR using *rplU* genes as reference gene. After infection for 4 days, 10 μg of PcrV was daily injected into the tissues surrounding the biofilm-infected catheters for three times. Infected tissues and catheters were harvested at day 8. Bacterial loads in infected tissues or catheters were determined by counting CFU **(C)** and production of TNFα and IL12 p40/70 was detected by ELISA assay **(D)**. F4/80^+^/iNOS^+^ and F4/80^+^/Arg1^+^ macrophages in tissues surrounding the biofilm-infected catheters were detected by immunofluorescence staining (**E**,**F**). For immunofluorescence staining, M1 macrophages were counterstained with FITC-conjugated anti-F4/80 and AF647-conjugated anti-iNOS antibodies. M2 macrophages were counterstained with FITC-conjugated anti-F4/80 and AF647-conjugated anti-Arg1 antibodies; cellular nuclei were stained with DAPI. One-way ANOVA (Tukey’s *post hoc*, **A**,**B**) or an unpaired Student’s *t* test (**C**,**D**) was used for statistical analysis. ^*^p < 0.05; ^**^p < 0.01; ^***^p < 0.001.

The reduced activation of host proinflammatory responses that allows *P. aeruginosa* biofilms to persist may be due to the inactivation of the T3SS, whereby components of the T3SS could be immunostimulatory. Previous research has shown that components of the *P. aeruginosa* needle tip complex, including PcrV, are involved in enhancing proinflammatory responses in mouse lung tissues following infection (Wangdi et al., 2010). Therefore, we speculated that *pcrV* gene expression is downregulated during *P. aeruginosa* biofilm infection, and the decrease in PcrV production in the milieu surrounding the infection accounts for the impaired activation of immune responses mediated by M2 macrophages following biofilm infection, which ultimately results in biofilm persistence. To verify this hypothesis, we first analyzed *pcrV* gene expression in *P. aeruginosa* biofilm-infected catheters *in vivo* by real-time quantitative PCR (RT-qPCR) analysis of three different *P. aeruginosa* reference genes. Compared to the immature biofilm bacteria (1 day post-infection), *pcrV* gene transcription was decreased both in the incompletely matured (4 days post-infection) and mature biofilm bacteria (7 days post-infection; Figure 2A). A similar trend was also observed in the *in vitro* static biofilm system (Figure 2B; *p* < 0.001). To further investigate the correlation between PcrV production and biofilm eradication, PcrV protein was subcutaneously injected into the tissues surrounding the biofilm-infected catheters. Bacterial burdens in PcrV-treated catheters (Figure 2C; *p* < 0.001) and surrounding tissues (Figure 2C; *p* < 0.05) were lower than those in the PBS-treated control group, whereas the levels of TNFα (Figure 2D; *p* < 0.01) and IL12 p40/70 (Figure 2D; *p* < 0.001) were elevated.

To explore the involvement of macrophages in PcrV-mediated elimination of biofilm bacteria, M1 and M2 polarization was analyzed in the macrophages associated with *P. aeruginosa* biofilm catheters-infected tissues following treatment with or without PcrV. The percentages of M1 macrophages (F4/80^+^/iNOS^+^) in the PcrV-treated groups were higher than those in the PBS-treated groups (Figure 2E), whereas the opposite trend was observed in M2 macrophages (F4/80^+^/Arg1^+^; Figure 2F). These results suggested that PcrV promotes the elimination of biofilm bacteria through polarization of macrophages toward an M1 phenotype.

### PcrV Is Involved in Skewing Macrophages Toward an M1 Phenotype

**Figure 3:**
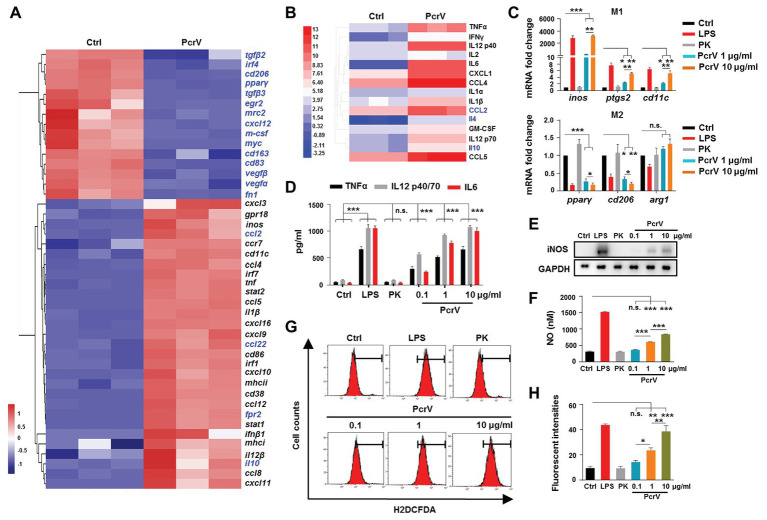
PcrV promotes macrophage M1 polarization. **(A)** Gene expression and **(B)** cytokine production in bone marrow-derived macrophages (BMDMs) treated with or without PcrV (10 μg/ml) for 24 h were analyzed by gene chip and protein chip, respectively. M1-related genes were marked in blank; M2-related genes were marked in blue. **(C)** Gene expression in BMDMs treated with LPS + IFNγ, hydrolyzed PcrV (PK), and PcrV (10 μg/ml) for 24 h was verified by RT-qPCR. **(D)** Levels of TNFα, IL12 p40/70, and interleukin 6 (IL6) in the culture supernatants of the treated BMDMs were assayed by ELISA assay. The production of iNOS **(E)** and reactive oxygen species (ROS; **G**,**H**) in Raw264.7 treated with LPS + IFNγ, PK, and PcrV for 6 h were detected by western blot and flow cytometry, respectively. **(F)** The concentration of nitric oxide (NO) in the culture supernatant of Raw264.7 treated with the indicated compound for 24 h was detected by NO detection kit. One-way ANOVA (Tukey’s *post hoc*, **C**,**D**,**F**,**G**) was used for statistical analysis. ^*^p < 0.05; ^**^p < 0.01; ^***^p < 0.001; n.s. indicates no significance.

Given that subcutaneous injection of PcrV promoted biofilm clearance and increased the percentages of M1-polarized macrophages around the infected catheter, we investigated the ability of PcrV to directly drive macrophage differentiation toward an M1 phenotype. To this end, we first evaluated the extensive inflammatory modulation effects of PcrV on BMDMs by gene and protein chip analysis. Gene chip analysis showed that PcrV treatment extensively upregulated macrophage M1 activation-related genes including proinflammtory cytokines (e.g., *tnf*, *il1β*, *il12*, and *il6*), chemokines (e.g., *cxcl3*, *cxcl9*, and *cxcl11*), bacterial killing molecules (*inos*), antigen presentation (e.g., MHCI, MHCII, and CD86), and others (e.g., *cd11c*, *ptgs2*, *egln3*, and *inhba*), whereas M2 activation-related genes, such as *cd206*, *pparγ*, *cd83*, and *egr2*, were downregulated (Figure 3A). Protein chip assays revealed that PcrV-primed BMDMs displayed increased production of macrophage M1 polarization-related cytokines (e.g., GM-CSF, TNFα, IFNγ, IL12 p40/70, and IL1α/β), chemokines (e.g., CXCL1 and CCL4/6), and IL2, which is responsible for T cell activation (Figure 3B). Despite the extensive upregulation of M1 markers following PcrV treatment, and decreased expression of M2 markers in BMDMs, some of the M2 molecules, such as *ccl2*, *ccl22*, IL4, and IL10 were also elevated in PcrV-primed BMDMs (Figures 3A,B), suggesting a balance of immune responses following PcrV treatment.

To observe the dose-dependent effects of PcrV on macrophages, BMDMs and the RAW264.7 cells were treated with different concentrations of PcrV. Similar to LPS-primed M1 macrophages, PcrV-pulsed BMDMs and RAW264.7 cells showed upregulated M1-related gene expression (*inos*, *ptgs2*, and *cd11c*; Figure 3C; Supplementary Figure S2A), increased production of iNOS (Figure 3E) and NO (Figure 3F), TNFα, IL12 p40/70, IL6 (Figure 3D; Supplementary Figure S2A), and ROS (Figures 3G,H), whereas expression of M2-related genes, including *cd206*, *pparγ* (Figure 3C; Supplementary Figure S2B), and *arg1* (Supplementary Figure S2B) was downregulated in a dose-dependent manner compared with the untreated‐ or hydrolyzed PcrV (PK)-treated groups. Taken together, these results suggested that PcrV plays a role in skewing macrophage differentiation toward an M1 phenotype.

### PcrV Repolarizes Macrophages From M2 to M1 Phenotype

**Figure 4:**
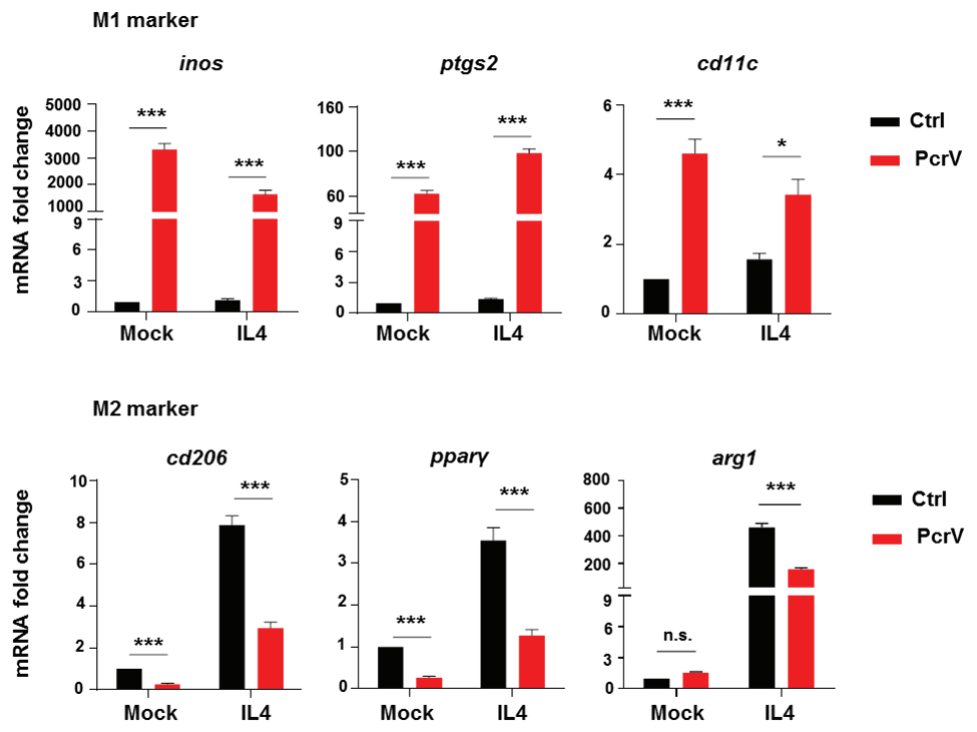
PcrV repolarizes macrophages from M2 to M1 phenotype. BMDMs pretreated with 20 ng/ml of IL4 for 12 h were primed by PcrV (10 μg/ml) for another 24 h. Macrophage M1 and M2-related genes were analyzed by RT-qPCR. An unpaired Student’s *t* test was used for statistical analysis. ^*^p < 0.05; ^***^p < 0.001; n.s. indicates no significance.

To evaluate the ability of PcrV to promote a macrophage switch from the M2 to M1 phenotype, BMDMs were pretreated with the M2 inducer IL4, before PcrV treatment. IL4 significantly increased the expression of M2-specific genes, including *cd206*, *pparγ*, and *arg1* in BMDMs (Figure 4); however, the IL4-mediated upregulation of these genes was abolished by PcrV treatment (Figure 4). Despite the anti-inflammatory conditions of macrophages pretreated with IL4, PcrV treatment significantly elevated the expression of proinflammatory M1-related genes in BMDMs, such as *inos*, *ptgs2*, and *cd11c* (Figure 4), demonstrating that PcrV plays a role in inducing transformation from the M2 to M1 phenotype.

### The Proinflammtory M1 Macrophages Polarized by PcrV Are Involved in Biofilm Elimination

**Figure 5:**
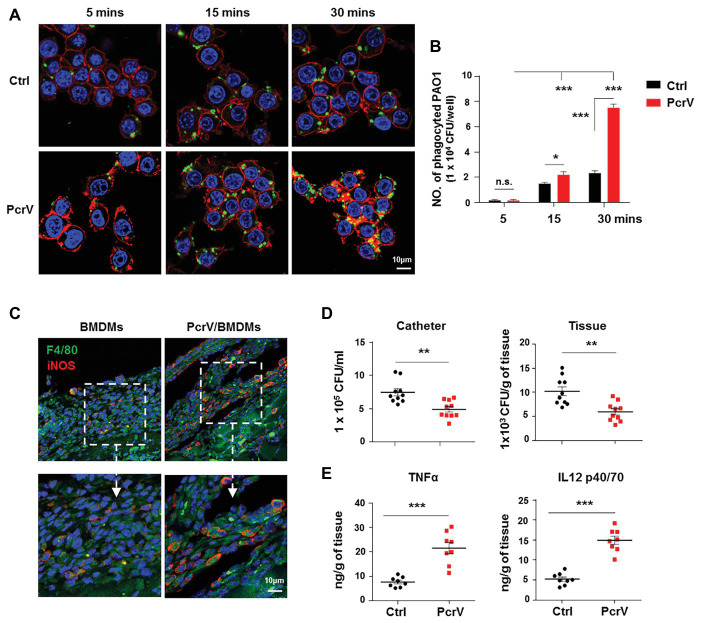
PcrV-primed macrophages display increased phagocytosis, bacterial killing efficacy, and induction of proinflammtory cytokines. Raw264.7 cells pretreated with or without PcrV (10 μg/ml) for 6 h were co-cultured with *P. aeruginosa* biofilms (MOI = 10) for the indicated time point. The phagocyted bacteria were detected by immunofluorescence staining **(A)** and CFU enumeration **(B)**. Cytoskeleton was labeled with phalloidin (red); PAO1 was visualized by FITC anti-PAO1 antibody (green); cellular nuclei were stained with DAPI (blue). BMDMs primed with or without PcrV (10 μg/ml) for 24 h were daily injected into the tissues surrounding biofilm-infected catheters after biofilm infection for 4 days. The infected tissues and catheters were harvested after the injection of PcrV for 3 days. F4/80^+^/iNOS^+^ macrophages in infected tissues **(C)**, bacterial burdens **(D)**, and production of TNFα and IL12 p40/70 **(E)** were analyzed by immunofluorescence staining, CFU enumeration, and ELISA, respectively. For immunofluorescence staining, M1 macrophages were counterstained with FITC-conjugated anti-F4/80 and AF647-conjugated anti-iNOS antibodies. M2 macrophages were counterstained with FITC-conjugated anti-F4/80 and AF647-conjugated anti-Arg1 antibodies; cellular nuclei were stained with DAPI. One-way ANOVA (Tukey’s *post hoc*, B) or unpaired Student’s *t* test (**D**,**E**) was used for statistical analysis. ^*^p < 0.05; ^**^p < 0.01; ^***^p < 0.001.

Considering that PcrV is involved in skewing macrophage toward the M1 phenotype, we further investigated the efficacy of PcrV-pulsed M1 macrophages in biofilm elimination both *in vitro* and *in vivo*. *In vitro* studies revealed that PcrV treatment significantly increased the phagocytosis ability of macrophages against biofilm bacteria in a time-dependent manner (Figures 5A,B; *p* < 0.001). Following injection of BMDMs pretreated with PcrV for 24 h into the tissues surrounding biofilm catheters *in vivo*, the percentage of F4/80^+^iNOS^+^ macrophages was higher in PcrV/BMDMs treated mice than that in mice treated with non-activated BMDMs (Figure 5C), suggesting that functional M1 macrophages were present at the site of infection. Analyses of bacterial burdens and tissue inflammation status revealed that PcrV-primed M1 macrophages reduced bacterial loads in catheters and infected tissues (Figure 5D; *p* < 0.01), whereas the production of TNFα and IL12 p40/70 was augmented (Figure 5E; *p* < 0.001). Collectively, these results suggested that PcrV-primed M1 macrophages are effective in accelerating biofilm clearance.

### MAPKs and NF-κB Signaling Pathways Play Dominant Roles in Promoting PcrV-Mediated Macrophage M1 Activation and Phagocytosis

**Figure 6:**
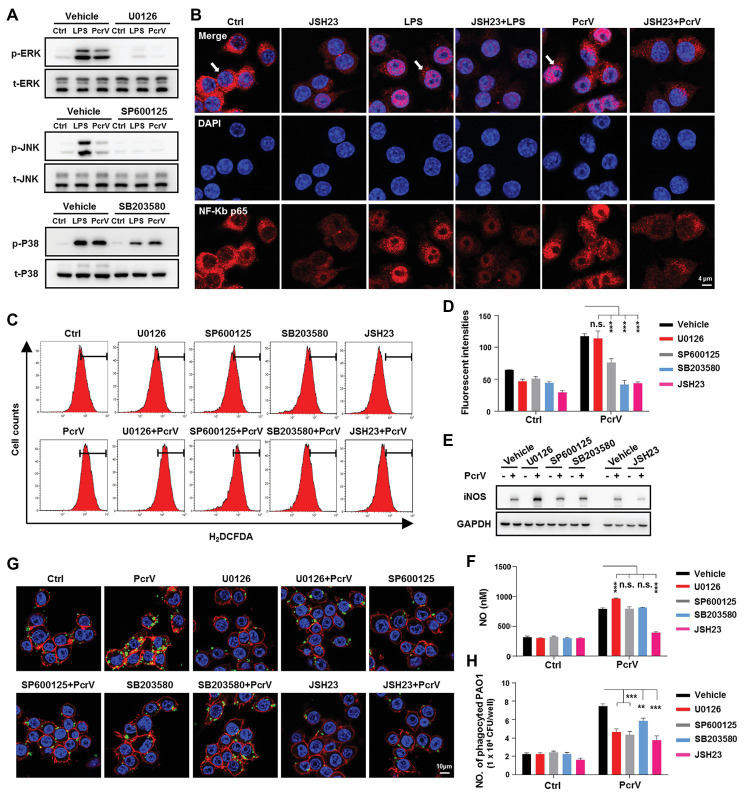
Mitogen-activated protein kinases (MAPKs) and NF-κB signaling pathways are involved in PcrV-mediated activation of M1 macrophages and increasing of phagocytosis. Raw264.7 cells pretreated with the corresponding inhibitors U0126 (5 μM), SP600125 (10 μM), SB203580 (5 μM), and JSH23 (15 μM) for extracellular signal-regulated protein kinase (ERK), c-Jun N-terminal kinase (JNK), p38 MAPKs, and NF-κB, respectively, were primed by LPS + IFNγ (named as LPS group) or PcrV (10 μg/ml) for 6 h. The total and phosphorylation levels of JNK, ERK, and p38 MAPKs were analyzed by western blot **(A)**. The cellular translocation of NF-κB was visualized by immunofluorescence staining **(B)**. NF-κB was labeled with AF647-conjugated anti-NF-κB p65 antibody (red); cellular nuclei were stained with DAPI (blue). ROS (**C**,**D**) and iNOS **(E)** production were analyzed by flow cytometry and western blot, respectively. **(F)** The concentration of NO in the culture supernatant of Raw264.7 treated with the indicated compound for 24 h was detected by NO detection kit. Raw264.7 pretreated with the corresponding inhibitors was primed by PcrV (10 μg/ml) for 6 h. The cells were then co-cultured with static PAO1 biofilms (MOI = 10) for 30 min. Phagocytosis was detected by immunofluorescence staining **(G)** and CFU enumeration **(H)**. Cytoskeleton was labeled with phalloidin (red); PAO1 was visualized by FITC anti-PAO1 antibody (green); cellular nuclei were stained with DAPI (blue). Unpaired Student’s *t* test was used for statistical analysis (**D**,**F**,**H**). ^**^p < 0.01; ^***^p < 0.001; n.s. indicates no significance.

MAPK and NF-κB signaling pathways are among the most extensively reported pathways that are involved in activating anti-bacterial immune responses, involving macrophage M1 polarization (Akhtar et al., 2019; Justino et al., 2020). Our results demonstrated that PcrV promoted the phosphorylation of extracellular signal-regulated protein kinase (ERK), c-Jun N-terminal kinase (JNK), and p38 MAPKs and IKBα (an indicator of NF-κB pathway activation) in RAW264.7 cells in a dose‐ and time-dependent manner (Supplementary Figures S3A,B). Further utilization of the corresponding signal pathway inhibitors U0126, SP600125, SB203580 for ERK, JNK, and p38 MAPKs, respectively, revealed that PcrV-mediated activation of the three pathways was successfully inhibited (Figure 6A). Since the nuclear translocation of NF-κB p65 results in the activation of NF-κB signaling pathway, we used JSH23, an inhibitor of NF-κB p65 nuclear translocation and transcriptional activity, to treat the cells. Given NF-κB p65 might regulate the transcriptional level of itself, the JSH23 treatment also lead to a massive reduction of the expression of NF-κB p65 in the JSH23 treated groups (Figure 6B). In spite of that, LPS or PcrV-mediated promotion of nuclear translocation of NF-κB p65 was impaired by JSH23 (Figure 6B). Analyses of the downstream cytokines, gene expression, and phagocytosis showed that the levels of TNFα, IL6 (Supplementary Figure S3C; *p* < 0.001), and ROS (Figures 6C,D), expression of the M1-related genes, *cd11c* and *ptgs*2 (Supplementary Figure S3D), and phagocytosis (Figures 6G,H) were reduced in PcrV-treated RAW264.7 in the presence of both MAPK and NF-κB inhibitors; however, ERK inhibition elevated iNOS (Figure 6E; Supplementary Figure S3D) and NO (Figure 6F; *p* < 0.001), while JNK and p38 inhibition did not alter NO production (Figure 6F). Meanwhile, MAPK inhibition did not elevate *pparγ* expression in RAW264.7 cells primed with PcrV (Supplementary Figure S3D). In contrast, JSH23 treatment reduced the levels of iNOS (Figure 6E; Supplementary Figure S3D; *p* < 0.001) and NO (Figure 6F; *p* < 0.001) and reversed the decrease in *pparγ* expression in PcrV-stimulated RAW264.7 cells (Supplementary Figure S3D; *p* < 0.05). Collectively, these results indicated that the MAPK and NF-κB signaling pathways are involved in PcrV-mediated regulation of macrophage M1 polarization and phagocytosis.

## Discussion

Bacterial biofilm formation on human tissues and implanted/indwelling devices provides a basis for persistent infections. The remodeling of host immune responses by biofilms following infection is one of the most important factors that benefit bacterial survival and chronicity of infection (Gonzalez et al., 2018; Campoccia and Mirzaei, 2019). Previous studies have revealed that *S. aureus* biofilms attenuated the production of proinflammatory cytokines/chemokines, including IL1β, TNFα, and CXCL2, as well as iNOS, and exacerbated bacterial biofilm infection (Thurlow et al., 2011). Chronic *P. aeruginosa* biofilm infections in cystic fibrosis patients are dominated by a Th2 response with increased and decreased levels of IL4 and IFNγ, respectively (Moser et al., 2000; Hartl et al., 2006), suggesting that *P. aeruginosa* biofilms persist by reducing host proinflammatory responses to infection. *In vitro* co-culture of *P. aeruginosa* biofilm matrix exopolysaccharides (EPS) and extracellular DNA (eDNA) with RAW264.7 cells induced a lower-grade inflammatory response than that induced by planktonic bacteria‐ or LPS-treated cells (Ramirez et al., 2019). In accordance with these reports, we have demonstrated that compared to planktonic bacteria, *P. aeruginosa* biofilms impaired the production of proinflammatory cytokines, as well as iNOS, while promoting the expression of the anti-inflammatory enzyme, Arg1, both *in vitro* and *in vivo*. Thus, our findings further confirm that *P. aeruginosa* biofilm-associated infection reduces proinflammatory responses to benefit biofilm bacterial survival.

Accumulating evidence demonstrates that the biofilm-mediated hijacking of host immune defense relies on several processes, such as interference in the release of antimicrobial peptides (AMPs), enzymes, ROS, RNS, and NO from leukocytes; impaired phagocytosis; and the recruitment of immunosuppressive cells, such as MDSCs (Campoccia and Mirzaei, 2019). In addition, studies have demonstrated that the re-education of M1 macrophages to an anti-inflammatory M2 phenotype is also involved in biofilm-mediated immune suppression (Thurlow et al., 2011; Hanke et al., 2013). In accordance with this phenomenon observed in *S. aureus*, we demonstrated that *P. aeruginosa* biofilms obstructed the host immune response by activation of M2 macrophages. Mechanistically, it has been shown that the cyclic di-AMPs, alpha-toxin (Hla), and leukocidin AB (LukAB; Scherr et al., 2015), released from *S. aureus* biofilms promote biofilm persistence by enhancing macrophage anti-inflammatory polarization or inhibiting phagocytosis (Gries et al., 2016). In *P. aeruginosa*, the biofilm formation-related exopolysaccharide alginates (Leid et al., 2005) and rhamnolipids (Alhede et al., 2014) have been implicated in the protection of biofilm bacteria against macrophage-mediated phagocytosis or have been shown to exert direct cytotoxic effect against macrophages. However, the mechanisms by which *P. aeruginosa* biofilms skew macrophage phenotypes to favor their survival following infection still remain obscure. Although excessive activation of *P. aeruginosa* T3SS during acute infection might aggravate inflammation-mediated tissue damage and immune dysfunction, activation of the host immune response by T3SS might also accelerate bacterial recognition and eradication by host immune cells (Galle et al., 2012; Klockgether and Tummler, 2017). However, during a chronic infection, bacterial T3SS is inactivated (Jain et al., 2008), which enhances the ability of bacteria to evade host immune recognition and clearance, ultimately resulting in biofilm persistence. Due to the reverse correlation between T3SS and biofilms (Kuchma et al., 2005), it is highly possible that regulatory elements that control T3SS inhibition/activation might also regulate biofilm persistence. In this study, we found that the T3SS protein PcrV, which is involved in enhancing proinflammatory polarization and phagocytosis of macrophages, is downregulated during *P. aeruginosa* biofilm formation *in vitro* and *in vivo*. The addition of exogenous PcrV or PcrV-pulsed M1 macrophages reversed M2 macrophage-mediated immune inhibition and increased biofilm bacterial elimination, indicating that the decreased expression of PcrV during biofilm formation might impair the M1 macrophage-mediated proinflammatory response and bacterial clearance, ultimately promoting biofilm persistence. Studies have demonstrated that *P. aeruginosa* T3SS genes, including *pcrV*, are negatively regulated in bacteria by the intracellular second messenger cyclic di-GMPs (Romling and Galperin, 2017) and the three-component SadARS regulatory system (Kuchma et al., 2005). Given that cyclic di-GMPs and the SadARS system are also involved in positively regulating *P. aeruginosa* biofilm formation (Kuchma et al., 2005; Ha and O’Toole, 2015; Sharp and Rietsch, 2019), it is likely that these factors are involved in modulation of PcrV-mediated regulation of biofilm persistence during infection *in vivo*. Considering that PcrV, which is involved in inducing macrophage M1 polarization and enhancing phagocytosis, is downregulated during biofilm infection, the addition of exogenous PcrV shows promise as a therapeutic strategy in patients with biofilm infections or chronic immune suppression.

Macrophage polarization is influenced by a variety of factors, such as different types of inflammatory cytokines/chemokines and infiltrated immune cells, as well as cell membrane and intracellular molecule-related mechanisms (Zhou et al., 2014). In this study, we demonstrated that PcrV-mediated polarization of M1 macrophages is through the activation of MAPK and NF-κB signal pathways. Similar to our findings, the bacterial pathogenicity associated molecular patterns (PAMPs) derived from Gram-negative bacteria, such as *Yersinia enterocolitica* LcrV (Sing et al., 2002), *Brucella abortus* cell-surface protein 31 (BCSP31) protein (Li et al., 2014), and *Vibrio cholerae* porin OmpU (Khan et al., 2015), have also been demonstrated to induce macrophage M1 polarization *via* MAPK and NF-κB signaling pathways. Peroxisome proliferator activated receptor (PPARγ), which is mainly expressed in adipose tissue and immune cells, is a member of the nuclear receptor superfamily of ligand-inducible transcription factors that regulate a variety of biological activities, including adipogenesis, lipid metabolism, and insulin sensitization, as well as inflammation (Janani and Ranjitha Kumari, 2015). PPARγ-mediated regulation of inflammation often leads to an inhibitory effect on the activation of immune cells, as well as the production of inflammatory factors through the suppression of signaling pathways, such as NF-κB and JNK/p38 signal pathways (Wang et al., 2018). In this study, we found that PcrV treatment significantly decreased the expression of *pparγ* gene in macrophages, suggesting that the impaired *pparγ* expression might exacerbate NF-κB and MAPK pathway-mediated inflammation in PcrV-treated macrophages.

In summary, this study reveals a role for PcrV in altering biofilm persistence of *P. aeruginosa*, and subsequently the potential therapeutic effects of treating *P. aeruginosa* biofilm infections with PcrV to reverse biofilm-mediated immune suppression.

## Data Availability Statement

The datasets generated for this study are available on request to the corresponding authors.

## Ethics Statement

The animal study was reviewed and approved by ethics committee of Army Medical University.

## Author Contributions

HY and KZ conceived and designed the experiments. HY, JX, JQ, XH, HS, QD, DL, RX, LJ, QL, QC, JP, and MW performed the experiments. HY, XR, and KZ analyzed the data. HY, XR, and KZ wrote the paper. All authors contributed to the article and approved the submitted version.

### Conflict of Interest

The authors declare that the research was conducted in the absence of any commercial or financial relationships that could be construed as a potential conflict of interest.
