# Supplementary material for: Type III Secretion Protein, PcrV, Impairs Pseudomonas aeruginosa Biofilm Formation by Increasing M1 Macrophage-Mediated Anti-bacterial Activities
Source: Front Microbiol. 2020 Aug 13;11:1971. doi: 10.3389/fmicb.2020.01971 (PMC7438568; doi:10.3389/fmicb.2020.01971)
Supplement: Supplementary file 1 [file Data_Sheet_1.DOCX]

**Supplementary Figures**

**
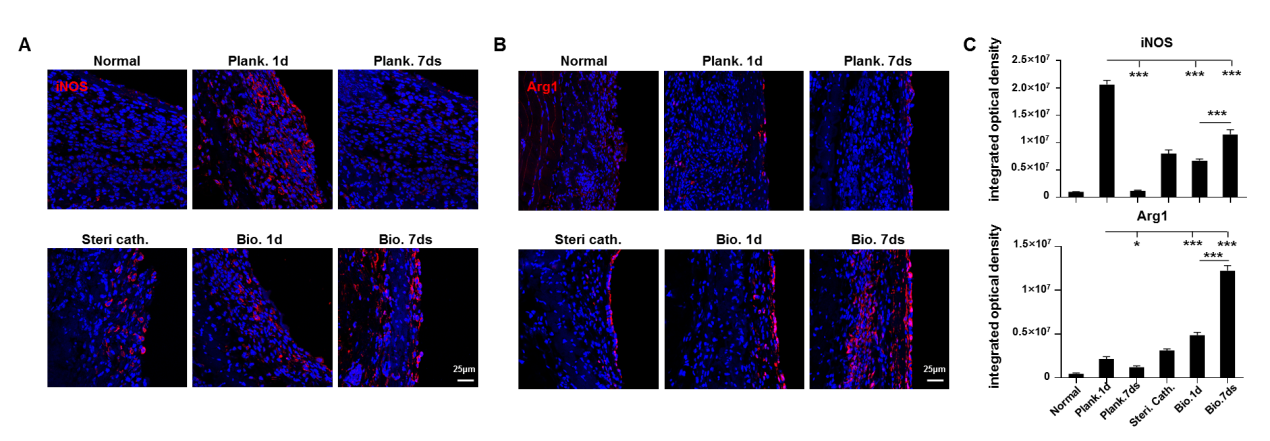
**

**FIGURE S1 *P. aeruginosa* biofilm catheter infection is associated with increased Arg1^+^ cells and decreased iNOS^+^ cells.** In planktonic bacteria-infected group, *P. aeruginosa* (1×10^5^ CFU) were subcutaneously into the flank of mice. For establishing of *P. aeruginosa* biofilm infection, *P. aeruginosa*-infected catheters (1×10^5^ CFU) were implanted subcutaneously into the flank of mice. The infected tissues and catheters were harvested at the indicated day. The PBS-treated normal tissues or sterile catheter-treated tissues were used as negative controls. The percentages of iNOS^+^ and Arg1^+^ cells in the infected tissues were determined by immunofluorescence staining. The iNOS^+^ (A) or Arg1^+^ (B) cells were stained with AF647-conjugated anti-iNOS or anti-Arg1 antibody, respectively; Cellular nuclei were stained with DAPI. (C) Quantification of the fluorescent intensity of iNOS^+^ and Arg1^+^ cells in each section was carried out by analyzing the integrated optical density using Image Pro Plus 6.0 software.


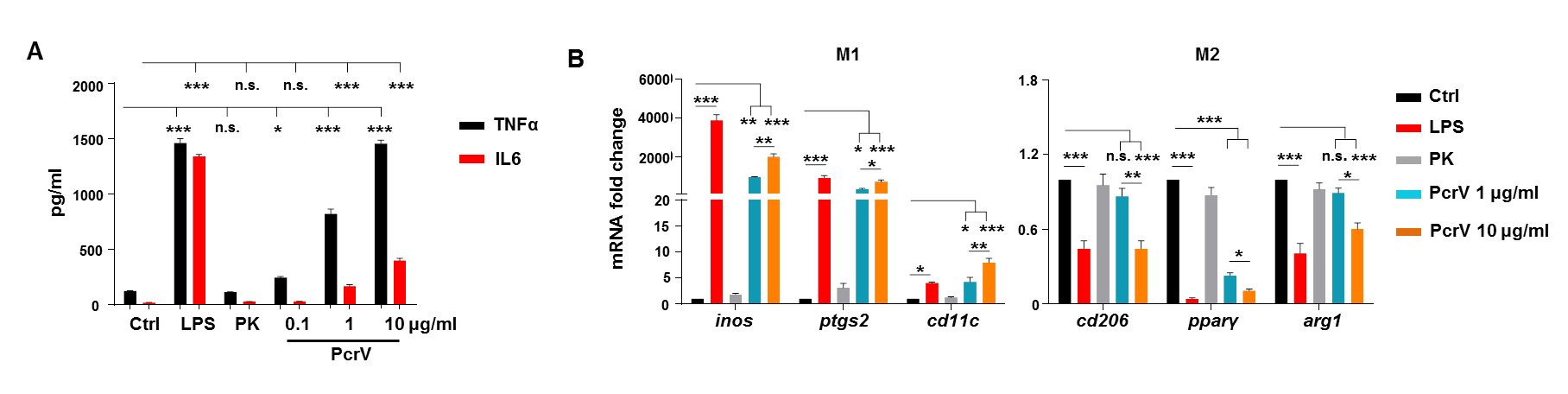


**FIGURE S2 PcrV promotes macrophage M1 polarization.** RAW264.7 was treated with 100 ng/ml LPS+50 ng/ml IFNγ, hydrolyzed PcrV (PK) or PcrV for 6 h. Production of TNFα and IL6 in the culture supernatants was assayed by ELISA (A). Gene expression was verified by RT-qPCR (B). One-way ANOVA (Tukey’s post hoc, A, and B) was used for statistical analysis. *, *P* < 0.05; **, *P* < 0.01; ***, *P* < 0.001. n.s. indicates no significance.


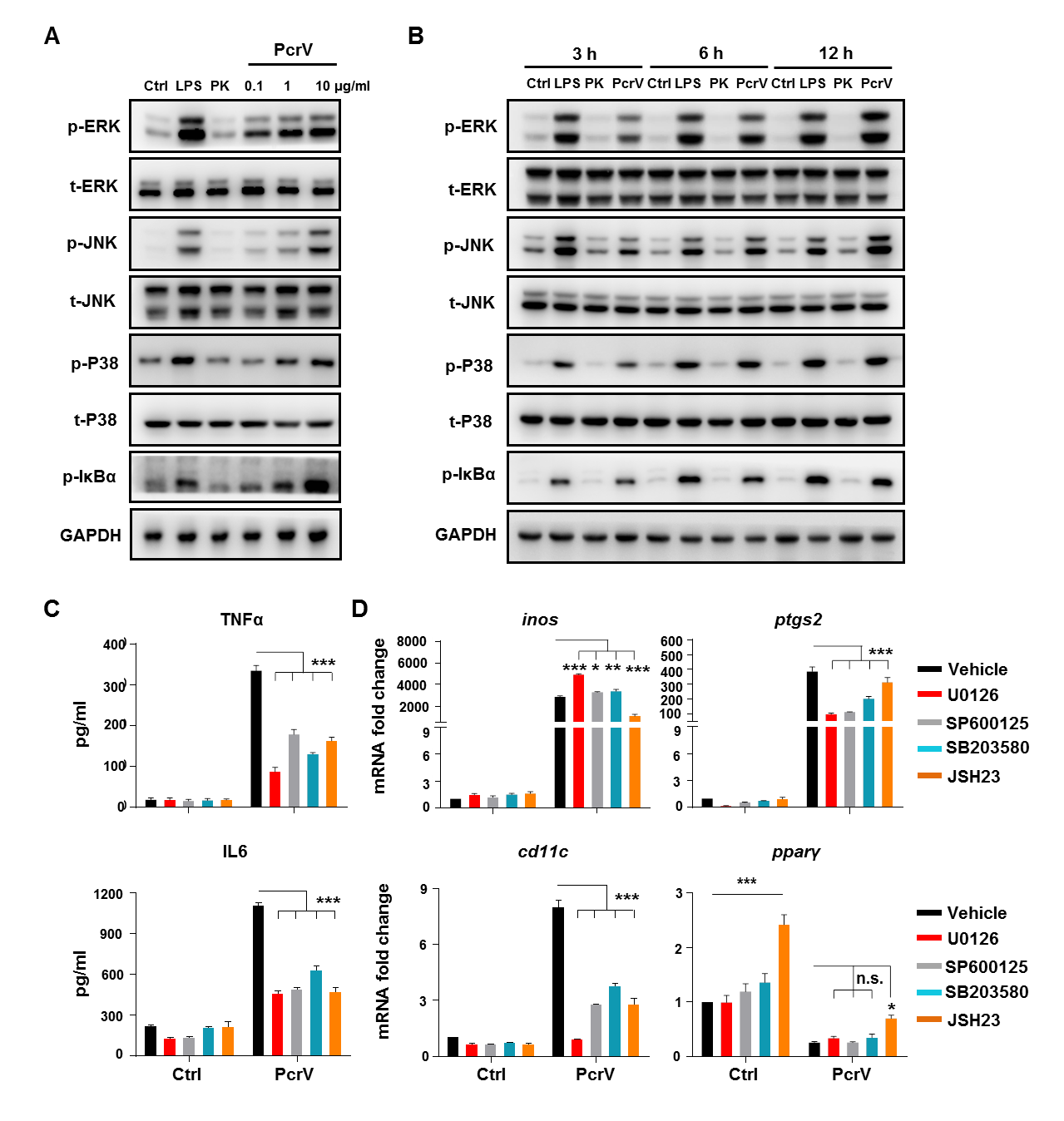


**FIGURE S3 MAPKs and NF-κB signaling pathways are involved in PcrV-mediated activation of M1 macrophages.** Raw264.7 was treated with PcrV at the indicated concentrations (A, for 6 h) and time points (B, PcrV 10μg/ml). The total and phosphorylation levels of JNK, ERK, and p38 MAPKs and IκBα were analyzed by western blot. Raw264.7 cells pretreated with the corresponding inhibitors U0126 (5 μM), SP600125 (10 μM), and SB203580 (5 μM) for ERK, JNK, and p38 MAPKs, respectively, were primed by PcrV (10 μg/ml) for 6 h. The production of TNFα and IL6 (C) and M1/M2-related genes (D) were analyzed by ELISA and RT-qPCR, respectively. An unpaired Student’s t test was used for statistical analysis (C and D). *, *P* < 0.05; **, *P* < 0.01; ***, *P* < 0.001; n.s. indicates no significance.

**TABLE S1 Primers used in this study**

| pcrV-FP | 5' GTGTCGGCCTATTTCAGCCT 3' |
| --- | --- |
| pcrV-RP | 5' TGATCTGCGACTGGATCACG 3' |
| iNOS-FP | 5' GTTCTCAGCCCAACAATACAAGA 3' |
| iNOS-RP | 5' GTGGACGGGTCGATGTCAC 3' |
| ptgs2-FP | 5' TGCACTATGGTTACAAAAGCTGG 3' |
| ptgs2-RP | 5' TCAGGAAGCTCCTTATTTCCCTT 3' |
| cd11c-FP | 5' AGAGCCAGAACTTCCCAACT 3' |
| cd11c-RP | 5' CTACCCGAGCCATCAATC 3' |
| cd206-FP | 5' GCAAGTGATTTGGAGGCT 3' |
| cd206-RP | 5' ATAGGAAACGGGAGAACC 3' |
| pparγ-FP | 5' TTTCAAGGGTGCCAGTTT 3' |
| pparγ-RP | 5' GAGGCCAGCATCGTGTAG 3' |
| ndk-FP | 5' ACCCTGTCCATCATCAAGCC 3' |
| ndk-RP | 5' GAACGGACGCTCTTTGTGC 3' |
| gyrB-FP | 5' CGCTGTCCCATGAACTACGC 3' |
| gyrB-RP | 5' GCCAGGATGTCCCAACTGAA 3' |
| rpoD-FP | 5' GGGCGAAGAAGGAAATGGTC 3' |
| rpoD-RP | 5' CAGGTGGCGTAGGTGGAGAA 3' |
| rplU-FP | 5' CGCAGTGATTGTTACCGGTG 3' |
| rplU-RP | 5' GGTAACCTTCGCACCTTCGA 3' |
| gapdh-FP | 5' CCTTCCGTGTTCCTACCC 3' |
| gapdh-RP | 5' AAGTCGCAGGAGACAACC 3' |

FP indicates forward primer; RP indicates reverse primer.
